# Multifactorial Modeling Reveals a Dominant Role of Wnt Signaling in Lineage Commitment of Human Pluripotent Stem Cells

**DOI:** 10.3390/bioengineering6030071

**Published:** 2019-08-15

**Authors:** Tiago P. Dias, Tiago G. Fernandes, Maria Margarida Diogo, Joaquim M. S. Cabral

**Affiliations:** 1 iBB—Institute for Bioengineering and Biosciences and Department of Bioengineering, Instituto Superior Técnico, Universidade de Lisboa, Av. Rovisco Pais, 1049-001 Lisbon, Portugal; 2 The Discoveries Centre for Regenerative and Precision Medicine, Lisbon Campus, Instituto Superior Técnico, Universidade de Lisboa, Av. Rovisco Pais, 1049-001 Lisbon, Portugal

**Keywords:** multiparameter, factorial design, Wnt signaling, TGFβ signaling, FGF signaling, human induced pluripotent stem cells, pluripotency and commitment

## Abstract

The human primed pluripotent state is maintained by a complex balance of several signaling pathways governing pluripotency maintenance and commitment. Here, we explore a multiparameter approach using a full factorial design and a simple well-defined culture system to assess individual and synergistic contributions of Wnt, FGF and TGFβ signaling to pluripotency and lineage specification of human induced pluripotent stem cells (hiPSC). Hierarchical clustering and quadratic models highlighted a dominant effect of Wnt signaling over FGF and TGFβ signaling, drawing hiPSCs towards mesendoderm lineages. In addition, a synergistic effect between Wnt signaling and FGF was observed to have a negative contribution to pluripotency maintenance and a positive contribution to ectoderm and mesoderm commitment. Furthermore, FGF and TGFβ signaling only contributed significantly for negative ectoderm scores, suggesting that the effect of both factors for pluripotency maintenance resides in a balance of inhibitory signals instead of proactive stimulation of hiPSC pluripotency. Overall, our dry-signaling multiparameter modeling approach can contribute to elucidate individual and synergistic inputs, providing an additional degree of comprehension of the complex regulatory mechanisms of human pluripotency and commitment.

## 1. Introduction

Human induced pluripotent stem cells (hiPSCs) have an incredible potential for regenerative medicine therapies, drug-screening and disease modeling [1,2,3]. Understanding pluripotency and controlling commitment is essential to take full advantage of hiPSC properties and to develop efficient protocols to induce hiPSC direct differentiation into the cell types of interest.

Human pluripotency is usually associated with a primed state, controlled by a complex balance between multiple signaling pathways that govern pluripotency maintenance and exit from pluripotency towards differentiation [4,5,6]. This state has been connected with a weak stability and a bias towards commitment resembling the mouse epiblast state [7,8], contrasting with the increased stability of the naïve pluripotent state [9,10,11].

FGF, TGFβ and Wnt signaling pathways are among the most important pathways controlling hiPSC fate [4,5,6]. These signaling pathways can be associated with pleiotropic effects, stimulating divergent cellular responses such as self-renewal and commitment [12,13,14]. For example, the combined effects of FGF signaling and TGFβ signaling are typically associated with hiPSCs self-renewal [4,15]. Individually, however, FGF signaling has been connected with both neuroectoderm inhibition [16] and activation [7,8,9]. On the other hand, TGFβ signaling results in SMAD2/3 activation, which is associated with mesendoderm lineage specification [17,18]. Importantly, Wnt/β-catenin signaling is associated with self-renewal in hiPSCs [4,19,20,21], in line with being essential to promote the naïve pluripotency state and inhibit epiblast transition [10,11,22,23]. However, during differentiation, Wnt signaling is also associated to self-renewal disruption and guidance of cells towards mesendoderm commitment [24,25]. Also noteworthy is the fact that Wnt signaling has a role in directing cells from neuroectoderm towards neural crest specification [25,26], and that it inhibits cardiac mesoderm specification [27,28] while promoting the epicardial cell fate [29]. Furthermore, these signaling pathways can be interconnected and influenced by multiple signals at different pathway nodes, resulting in synergistic or antagonistic effects that can shift commitment towards specific lineages [30,31,32,33]. Thus, complex and undefined culture systems with multiple signaling inputs, often using conditioned media or serum, can provide a signaling overload, contributing to divergent and pleiotropic responses, that can mask the true impact of each signaling input. Development of a multiparameter approach with a controlled signaling environment can allow to fully discern the multiple singular and cooperative contributions of each signaling input allowing the identification of synergistic and antagonistic effects [34].

We previously used a multifactorial analysis approach that revealed a significant contribution of Wnt signaling to mESC pluripotency under physiological oxygen tensions [34]. Here, we use a dry-signaling multiparameter approach consisting of a full factorial design, combining the activation of Wnt, FGF and TGFβ signaling in hiPSCs cultured in a simple and well-defined culture system. Hierarchical clustering and quadratic models for human pluripotency and lineage commitment were designed and highlighted a Wnt signaling dominance with or without the presence of FGF and TGFβ inputs. Synergistic effects were observed between Wnt and FGF signaling by the pluripotency, ectoderm and mesoderm models. In addition, FGF and TGFβ signaling contributed negatively to the ectoderm model without a significant contribution for the pluripotency model, suggesting that a balanced inhibitory effect is promoting hiPSC pluripotency maintenance.

## 2. Materials and Methods

### 2.1. Human Induced Pluripotent Stem Cell Culture

In this work, the hiPSC cell line iPS-DF6-9-9T.B, purchased from WiCell Bank, was mainly used. This cell line is vector free and was derived from foreskin fibroblasts with a karyotype 46, XY. Both the hiPSC cell line F002.1A.13 provided by TCLab (Tecnologias Celulares para Aplicação Médica, Unipessoal, Lda.) that was generated using a retroviral system and the hiPSC line Gibco™ (Thermo Fisher Scientific, Waltham, MA, USA) derived from CD34^+^ cells of healthy donors were used to validate results as described in the different sections and figure legends.

Maintenance of hiPSC culture was performed using an mTeSR1 medium (STEMCELL Technologies, Vancouver, BC, Canada) in 6-well tissue culture plates coated with Matrigel (BD Biosciences, San Jose, CA, USA) and diluted 1:30 in DMEM/F12. The medium was changed daily. Human iPSC passaging was performed using an EDTA (Thermo Fisher Scientific, Waltham, MA, USA) solution diluted in PBS at a concentration of 0.5 mM. Cells were incubated for 5 min with EDTA at room temperature and flushed with culture medium. For maintenance cultures, splits from 1:3 to 1:8 were usually performed. For cell counting, a sample of 100 µL was incubated in 400 µL of Accutase for 7 min at room temperature and samples were diluted 1:2 in Trypan Blue (Thermo Fisher Scientific, Waltham, MA, USA) for counting using a hemocytometer. Culture photos were obtained using a Leica DMI 3000B microscope (Leica Microsystems GmbH, Wetzlar, Germany) and a digital camera Nikon DXM 1200 (Nikon, Tokyo, Japan).

### 2.2. Full Factorial Design

A 3^3^ full factorial design consisting of 27 culture conditions, corresponding to different concentrations of three different soluble factor activators of FGF, TGFβ and Wnt signaling (FGF2, TGFβ and CHIR, respectively), as well as three concentration levels (0, 1/3 and 1), was performed using E6 medium (Thermo Fisher Scientific, Waltham, MA, USA) as the basal medium. FGF2 (PeproTech, Rocky Hill, NJ, USA) concentration levels ranged from 0, 35 ng/mL to 100 ng/mL; TGFβ1 (PeproTech, Rocky Hill, NJ, USA) concentration levels ranged from 0, 0.7 ng/mL to 2 ng/mL; and CHIR99021 (Stemgent, Cambridge, MA, USA) concentration levels ranged from 0, 2 µM to 6 µM (Table 1). Three blocks of 9 culture conditions (samples) were performed each time with mTeSR1, E8 and E6 as controls. Cells were collected by EDTA Enzyme-free passaging and were seeded at 37,500 cells/cm^2^ using an mTeSR1 medium, to guarantee that the results of the study would not be affected by cell confluence. Conditions were exposed to the respective cocktail after 24 h and fresh supplemented medium changed every 24 h for 4 consecutive days of exposure. Fresh medium was prepared every day and supplemented with the cytokines and small molecules prior to medium change. After 4 days of exposure, cells were singularized using Accutase for 7 min, centrifuged, and a sample counted to evaluate cell number fold increase (FI) using trypan blue. Cells were washed with PBS, centrifuged, and the cell pellets were stored at −80 °C to perform real-time PCR afterwards.

### 2.3. Human iPSC-Cardiomyocyte (hiPSC-CM) Differentiation

Human iPSCs were seeded at a density of 1 × 10^5^ cells/cm^2^ and maintained in pluripotency conditions with daily medium changes. When confluence reached percentages around 95%, hiPSC cardiac differentiation was induced following the Wnt signaling modulation protocol previously described by Lian et al. [35]. Experiments were performed using 1 µM or 6 µM of the GSK3β inhibitor CHIR99021 (Stemgent, Cambridge, MA, USA) at day 0 and with or without 5 µM of the Wnt signaling inhibitor IWP4 (Stemgent, Cambridge, MA, USA) at day 3. Cells were collected and analyzed at day 15 of differentiation.

### 2.4. Human iPSC-Neural Differentiation

Human iPSCs were seeded at a density of 2 × 10^5^ cells/cm^2^ using E8. For E6 differentiation, after overnight growth, the medium was changed to E6 as previously described by Lippmann et al. [36]. For dual SMAD Inhibition-based neural induction, after cultures were nearly confluent, the medium was changed to 1:1 N2/B27 media supplemented with 10 μM SB431542 (Stemgent, Cambridge, MA, USA) and 100 nM LDN193189 (Stemgent, Cambridge, MA, USA), as previously described [37,38]. For both protocols, the medium was changed daily, and cells were collected and analyzed at day 12 of differentiation.

### 2.5. Flow Cytometry

Cells were washed with PBS, singularized and fixed using 2% (*v*/*v*) PFA for 20 min at room temperature. Cells were centrifuged and resuspended in 90% (*v*/*v*) cold methanol, incubated for 15 min at 4 °C. Samples were then washed 3 times using a solution of 0.5% (*v*/*v*) BSA in PBS (FB1). Primary antibody Cardiac Troponin T (CTNT) monoclonal mouse IgG antibody (Thermo Fisher Scientific, Waltham, MA, USA, Clone 13-11, dilution 1:250) or Primary antibody T/Brachyury polyclonal goat IgG antibody (R&D Systems, dilution 1:20) were diluted in FB1 plus 0.1% (*v*/*v*) Triton (FB2) and incubated for 1 h at room temperature. Cells were then washed and the cell pellet resuspended with the secondary antibody goat anti-mouse Alexa-488 (Thermo Fisher Scientific, Waltham, MA, USA) for CTNT or secondary antibody donkey anti-goat Alexa-488 for T/Brachyury (Thermo Fisher Scientific, Waltham, MA, USA), both diluted 1:1000 in FB2 and incubated for 30 min in the dark. Finally, cells were washed twice and cell pellets were resuspended in 500 µL of PBS and analyzed in a FACSCalibur flow cytometer (Becton Dickinson, Franklin Lakes, NJ, USA). Data were analyzed using the software “Flowing Software” at http://www.flowingsoftware.com (version 2.5).

### 2.6. Immunofluorescence Staining

Cells were fixed with 4% (*v*/*v*) PFA for 15 min, washed with PBS and incubated with blocking solution (10% *v*/*v* NGS, 0.1% *v*/*v* Triton-X in PBS) for 1 h. After incubation, for hiPSC-CM differentiation, Cardiac Troponin T (CTNT) monoclonal mouse IgG antibody (Thermo Fisher Scientific, Waltham, MA, USA, Clone 13-11) was diluted 1:250 in staining solution (5% *v*/*v* NGS, 0.1% *v*/*v* Triton-X in PBS) and incubated for 2 h at room temperature. For hiPSC-Neural commitment, NESTIN monoclonal mouse IgG antibody (R&D Systems, Minneapolis, MN, USA) and PAX6 polyclonal rabbit IgG antibody (Covance, Princeton, NJ, USA) were used both diluted 1:1000 in staining solution and incubated for 2 h at room temperature. After washing with PBS, secondary antibodies goat anti-mouse IgG Alexa-546 and goat anti-rabbit IgG Alexa-488 (Thermo Fisher Scientific, Waltham, MA, USA) were diluted 1:500 in staining solution and incubated for 1 h at room temperature. Samples were then washed 2 times with PBS, incubated for 2 min with 3 µg/mL of DAPI diluted in PBS, washed again 3 times, and stored at 4 °C. Samples were analyzed using a fluorescence optical microscope (Leica DMI 3000B, Leica Microsystems GmbH, Wetzlar, Germany) and a digital camera (Nikon DXM 1200, Nikon, Tokyo, Japan). Images were processed using ImageJ/Fiji (http://fiji.sc) [39] and PAX6^+^ cells were quantified using CellProfiler (Broad Institute, Cambridge, MA, USA).

### 2.7. Real-Time PCR

RNA from each condition and controls was extracted using the High Pure RNA Isolation Kit (Roche, Basel, Switzerland) following the instructions provided with the Kit. RNA was quantified using a nanodrop, and 1 µg of RNA was converted to cDNA using the High Capacity cDNA Reverse Transcription Kit (Thermo Fisher Scientific, Waltham, MA, USA) following the instructions provided with the kit. Relative gene expression was evaluated using 10 ng of cDNA, 250 µM of each primer (Table S1) and using the Fast SYBR Green Master Mix (Thermo Fisher Scientific, Waltham, MA, USA) with an annealing temperature set to 60 °C. Melting curves were performed at the end to assess if primers were amplifying only the correct amplicon. Values were treated following the 2^−ΔΔCT^ method. *GAPDH* gene expression was used as endogenous control and relative expression was calibrated for each gene using mTeSR1 gene expression values. For hiPSC-CM differentiation, relative expression was calibrated using day 0 of differentiation.

For hiPSC-Neural commitment, real-time PCR was performed using the TaqMan Gene Expression Assay (Thermo Fisher Scientific, Waltham, MA, USA) for the genes *OCT4*/*POU5F1* (Hs00999634_gH), *NANOG* (Hs02387400_g1), *PAX6* (Hs00240871_m1), *SOX1* (Hs01057642_s1) and *GAPDH* (Hs02758991_g1). *GAPDH* gene expression was used as endogenous control and relative expression was calibrated using day 0 of differentiation.

### 2.8. Panels and Scores

Relative expression values were normalized using the minimum and maximum value obtained for each gene. Then, panels for pluripotency (*OCT4* and *NANOG*), ectoderm (*FGF5*, *PAX6* and *P75*), mesendoderm (*MIXL1* and *T*), mesoderm (*NKX2.5* and *MESP1*) and endoderm (*SOX17* and *PDX1*) were created by averaging the expression value of each gene. Then, scores for pluripotency and for each lineage were empirically calculated as follows:

(1) $$\begin{array}{c} Pluripotency\;Score=1.5\times Pluripotent\;Panel-0.25\times Ectoderm\;Panel\\ -0.25\times Mesendoderm\;Panel-0.5\times Mesoderm\;Panel-0.5\times Endoderm\;Panel, \end{array}$$

(2) $$\begin{array}{c} Ectoderm\;Score=1.75\times Ectoderm\;Panel-0.25\times Pluripotent\;Panel-\\ 0.5\times Mesendoderm\;Panel-0.5\times Mesoderm\;Panel-0.5\times Endoderm\;Panel, \end{array}$$

(3) $$\begin{array}{c} Mesendoderm\;Score=Mesendoderm\;Panel+0.25\times Endoderm\;Panel\\ +0.25\times Mesoderm\;Panel-0.5\times Ectoderm\;Panel-Pluripotent\;Panel, \end{array}$$

(4) $$\begin{array}{c} Endoderm\;Score=2\times Endoderm\;Panel+0.5\times Mesendoderm\;Panel-\\ 0.5\times Mesoderm\;Panel-Ectoderm\;Panel-Pluripotent\;Panel, \end{array}$$

(5) $$\begin{array}{c} Mesoderm\;Score=2\times Mesoderm\;Panel+0.5\times Mesendoderm\;Panel-\\ 0.5\times Endoderm\;Panel-Ectoderm\;Panel-Pluripotent\;Panel. \end{array}$$

The main results showed in this study using scores were not changed when panels or individual gene expression were used. Nevertheless, scores helped to clarify the true effect of signal combinations, leading to more robust, statistically significant models.

### 2.9. Hierarchical Clustering and PCA

Hierarchical clusters and principal component analysis (PCA) were performed using Clustvis, a web tool based on R [40]. Clusters were obtained using Pearson correlation and average linkage. PCA were obtained using the Clustvis default SVD imputation.

### 2.10. Full Factorial Design Models and Statistical Analysis

A model for each score was created using Statistica Software. Models were obtained by fitting the data to a full quadratic model (linear, quadratic and two-way interactions) with centered and scaled polynomials, as follows:

(6) $$Y_{i}=\beta_{0}+\beta_{1}\left[X_{1}\right]+\beta_{11}{\left[X_{1}\right]}^{2}+\beta_{2}\left[X_{2}\right]+\beta_{22}{\left[X_{2}\right]}^{2}+\beta_{3}\left[X_{3}\right]+\beta_{33}{\left[X_{3}\right]}^{2}\;+\beta_{12}\left[X_{1}\right]\left[X_{2}\right]+\beta_{13}\left[X_{1}\right]\left[X_{3}\right]+\beta_{23}\left[X_{2}\right]\left[X_{3}\right]$$

where *Y_i_* corresponds to the specific score; *β*_0_ is the intersect coefficient; *β*_1_, *β*_2_ and *β*_3_ are the coefficients correspondent to the linear main effects; *β*_11_, *β*_22_ and *β*_33_ are the quadratic coefficients and *β*_12_, *β*_13_ and *β*_23_ are the coefficients for factor interactions. The full factorial design with three replicates of Sample 1 (E6) resulted in a total of 28 degrees of freedom. Statistical significance for each model was assessed by ANOVA using Fisher’s statistical test, in which factors with *p*-values lower than 0.05 were considered to have a statistically significant contribution to the model [34,41]. Models were not further refined by discarding non-statistically significant factors. Nevertheless, R^2^-adjusted (R^2^-Adj), a modified version of R^2^, is also showed for every model. R^2^-Adj compares the explanatory power of the regression models calculated with many prediction factors by discarding factors that do not significantly improve model prediction, and therefore helps to assess the true quality of the model.

## 3. Results

### 3.1. Full Factorial Analysis in a “Dry-signaling” Culture System

**Figure 1:**
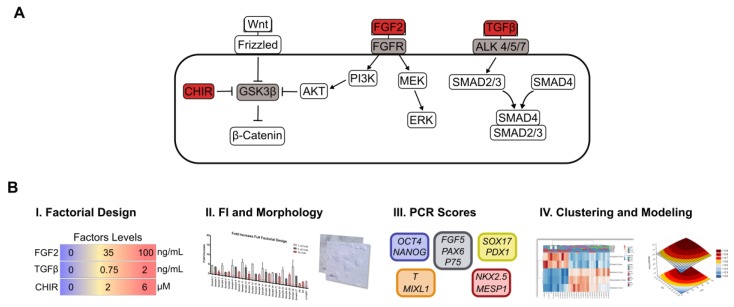
Schematic representation of the experimental framework used in this study. (**A**) A multiparameter approach was designed to reveal FGF, TGF/Nodal and Wnt signaling synergistic impact on human pluripotency and exit towards differentiation. (**B**) Multiparameter methodology performed. (i) A full factorial design combining 3 factors and 3 concentration levels for each factor was performed using E6 and Vitronectin as a dry-signaling culture system. Cells were exposed to the respective molecule cocktails for 4 days, with the medium changed daily. (ii) Proliferation and morphology were assessed for all 27 conditions plus mTeSR1 and E8 at the end of the 4-day culture. (iii) Real-time PCR was performed for each panel, and scores for pluripotency, ectoderm, mesendoderm, mesoderm and endoderm were calculated for each condition. Finally, (iv) scores obtained for each condition were hierarchical clustered and fitted to full quadratic models for each score.

To expose the impact of FGF signaling, TGF/Nodal signaling and Wnt signaling in human pluripotency and exit towards differentiation, a full factorial design was conceived to detect dual signaling roles by combining three concentration levels of each signaling input: Zero, lower activation (1/3 of higher activation) and higher activation, using E6/VTN [15], a dry-signaling system, as the basal culture medium (Figure 1). When compared with the E8 formulation [15], the E6 medium has only insulin as a principal signaling input, eliminating from its formulation FGF2 and Nodal/TGFβ. The experimental design covered 27 different conditions (Table 1). In addition, three replicates of each E6 basal media (Sample 1), mTeSR and E8 experiments were performed as controls.

In our multiparameter approach, FGF pathway was modulated using FGF2 at concentrations of 0, 35 and 100 ng/mL. Both TeSR and E8 medium use 100 ng/mL of FGF2 to maintain hiPSC pluripotency [15,42]. At this concentration and higher, a plateau of maximal activity is observed for downstream FGF signaling targets such as ERK and FRS-2 [12]. In fact, maximum activation of both downstream targets can be observed at 10 ng/mL, which can contribute to the pleiotropic behavior of FGF signaling [12]. In addition, TGF pathway was modulated using TGFβ1 at concentrations of 0, 0.7 and 2 ng/mL. E8 medium uses 1.74 ng/mL of TGFβ1 to maintain hiPSC pluripotency, although this concentration also has an impact in fibroblast proliferation and can inhibit hiPSC reprograming [43]. In TeSR, 0.6 ng/mL of TGFβ1 has a mild contribution to maintain pluripotency by directly targeting NANOG [17,42]. TGFβ1 at a concentration of 1 ng/mL seems to be enough to plateau maximum expression of downstream targets such as SMAD3 and release of IL-6 and CXCL8 [13]. Lastly, the Wnt pathway was modulated using the small chemical inhibitor CHIR99021 (CHIR) at concentrations of 0, 2 and 6 µM, which inhibits GSK3β leading to canonical Wnt signaling activation [44]. CHIR is one of the most potent and specific GSK3β inhibitors in vitro and seems to not significantly affect other kinases [44,45,46,47]. A concentration of 6 µM of CHIR is commonly used to promote hiPSC exit from pluripotency towards mesendoderm [28,35]. Lower concentrations, usually up to 2 µM, are found to be involved in self-renewal of human naïve PSCs [10], while 3 µM in the presence of dual SMAD inhibitors can induce hPSC neural crest differentiation [48].

**Figure 2:**
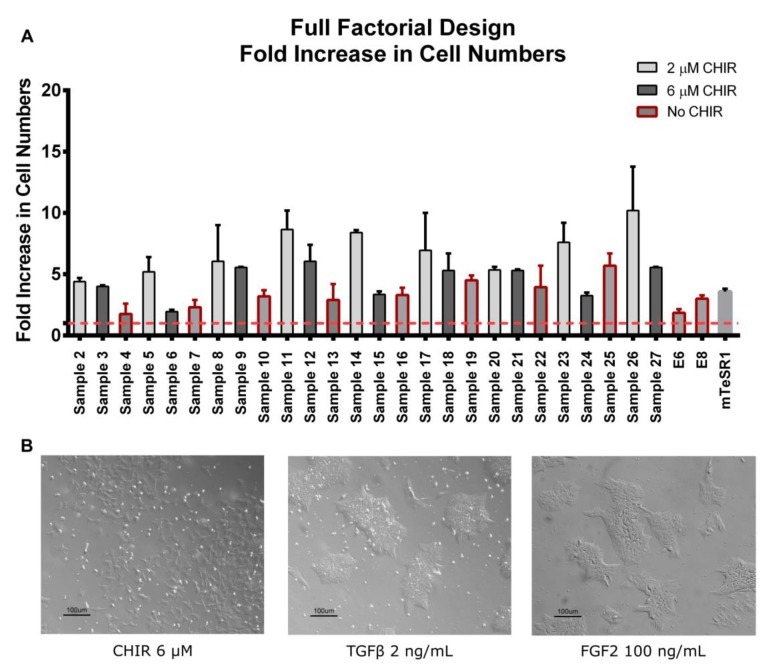
Human iPSC fold increase in total cell numbers and morphology of full factorial design conditions and controls. (**A**) Cell fold increase of all full factorial design conditions and controls. Red-dotted line marks the minimal threshold for fold increase achievement (FI = 1). In general, medium cocktails supplemented with CHIR showed higher cell fold increases when compared to cocktails without CHIR (highlighted in red). Error bars, standard error of the mean (SEM), *n* = 2. *p*-value < 0.01 by one-way ANOVA. (**B**) Typical morphology of cultures with CHIR addition compared to cultures without CHIR addition after 72 h of exposure to signaling inputs. Cultures without CHIR retained typical pluripotent colony morphology when compared to E8 or mTeSR1, while CHIR supplementation showed no colonies, which is associated with a more committed phenotype. See Table 1 for detailed concentrations of samples.

Each condition of the full factorial design was assessed by analyzing the overall fold increase in total cell numbers, colony morphology and by real-time PCR, allowing the attribution of scores to each condition and the assessment of the data by clustering and modeling tools (Figure 1B). To assess the effect of each input, human iPSCs were seeded in VTN using mTeSR1. After 24 h, hiPSCs were exposed to the respective cocktail of signaling inputs using E6 as basal media. Exposure was performed for 4 days, changing the media every 24 h, to assess if specific cocktail combinations contributed to maintain pluripotency or guided cells to differentiation. Cell fold increase for all experimental conditions (samples) was evaluated after 5 days in culture (Figure 2A). All experimental conditions promoted cell growth, although, in general, cells exposed to media cocktails without CHIR presented a lower cell growth compared to conditions with CHIR supplementation. This result was consistent with the cell morphology observed, with cells exposed to cocktails containing CHIR showing a more differentiated-like phenotype at day 4 when compared with more well-defined compact colonies, typically associated with the pluripotent state, for cells without exposure to CHIR (Figure 2B). These cell morphology changes were observed gradually with increased CHIR exposure time (Figure S1). The effect of Wnt activation in colony morphology is in clear contrast with the effect of CHIR, at lower concentrations, commonly observed for mice or human cells in the naïve state of pluripotency with round-cells organized in a more compact and multilayer-like colony morphology [9,11,34,49].

### 3.2. Pluripotency and Lineage Specification Evaluation Using Hierarchical Clustering and Principal Components Analysis

To assess the effect on cells exposed to each signaling input, real-time PCR was performed to analyze the expression of a set of genes (Table S1), corresponding to pluripotency and different lineage markers, whose expression levels could indicate if pluripotency was maintained or cells started to commit towards a specific lineage upon exposure to the different molecular cocktails. For the pluripotency panel, OCT4 and NANOG were selected, since both form the pluripotency core with SOX2, with OCT4 being enough to maintain and induce pluripotency [50] and NANOG being a sensible marker and gatekeeper of the pluripotent state [51,52,53]. Ectoderm panel was constituted by FGF5, a post-implantation primitive ectoderm marker [54]; PAX6, an early marker of neuroectodermal differentiation [55]; and P75, a neural crest cell marker [26]. Mesendoderm panel was composed by the primitive streak genes T/Brachyury, essential for primitive streak formation and mesendoderm differentiation, and MIXL1, a mesendoderm morphogen appearing at later stages of differentiation [56,57,58]. The endoderm panel was constituted by SOX17, a sensitive definitive endoderm marker [18,31], and PDX1, a foregut endoderm marker and regulator of pancreas specification [59]. The mesoderm panel was defined by MESP1, an early mesoderm marker that contributes to the specification of multiple mesoderm lineages in a context-dependent manner [60], and NKX2.5, a cardiac mesoderm marker expressed upon cardiac crescent formation [61].

**Figure 3:**
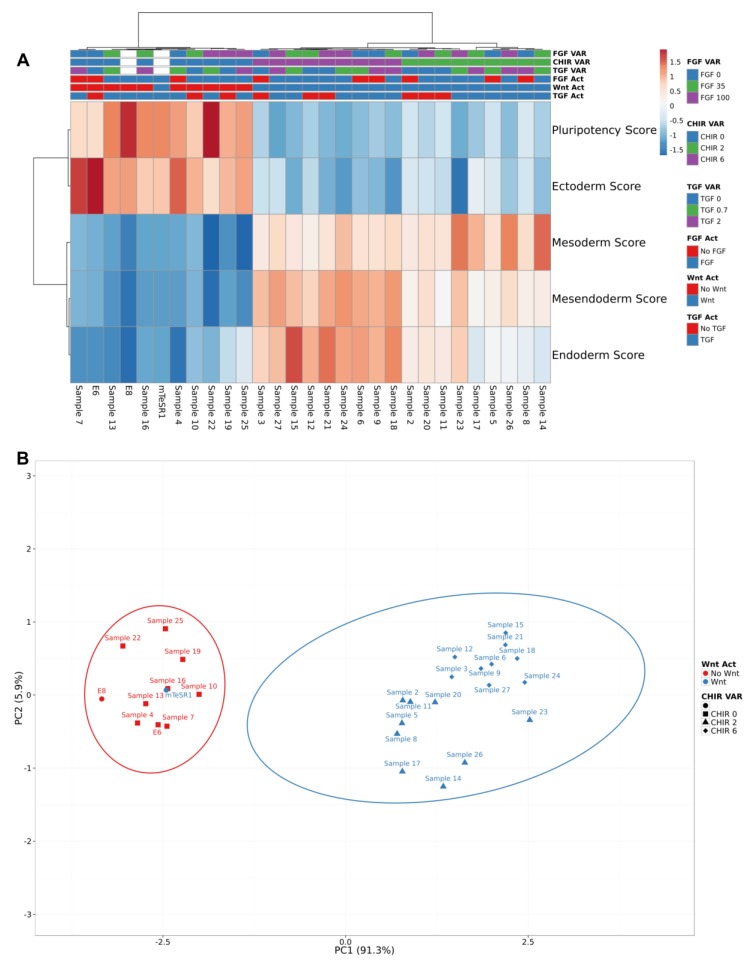
Hierarchical clustering and PCAs of full factorial design scores revealed two main clusters concordant with the presence or absence of CHIR. (**A**) Two main clusters were observed: Wnt activation, characterized by higher scores to mesendoderm lineages; and No Wnt activation, characterized by higher pluripotency and ectoderm scores. Clustering was performed using Pearson correlation and average linkage. Samples were labelled using signaling activation and no activation (FGF Act, Wnt Act and TGF Act; red and blue) and concentration variation (FGF VAR, CHIR VAR and TGF VAR; blue, green and purple). (**B**) The two main principal components together explained 97.2% of total data set variance. PC1 variance (91.3% of data set) corresponds to Wnt modulation. Only mTeSR1 clustered within the “No Wnt” group.

To emphasize the main path that hiPSCs were following after exposure to the signaling cocktails, scores to each lineage commitment and pluripotency were attributed to each sample. This data was hierarchically clustered using Pearson correlation and average linkage, resulting in two main clusters mainly explained by the presence or absence of Wnt signaling activation (Figure 3A). Cocktails exposing hiPSCs to CHIR clustered together and led to higher mesendoderm, endoderm and mesoderm scores, while conditions without CHIR clustered together and led to higher pluripotency and ectoderm scores. In addition, PCAs show that 91.3% of data variability (PC1) rely on Wnt signaling variation (Figure 3B). The exception was hiPSCs exposed to mTeSR1, which has LiCl (0.1 mM), a Wnt activator [42], and registered higher pluripotency and ectoderm scores, with lower mesendoderm, mesoderm and endoderm scores. These results seem to further highlight colony morphology observations: Wnt signaling activation was imposing hiPSCs to exit pluripotency and to commit towards mesendoderm lineages with or without FGF and TGF activation.

### 3.3. Full Quadratic Models for the Pluripotency and Ectoderm Lineage Scores

**Figure 4:**
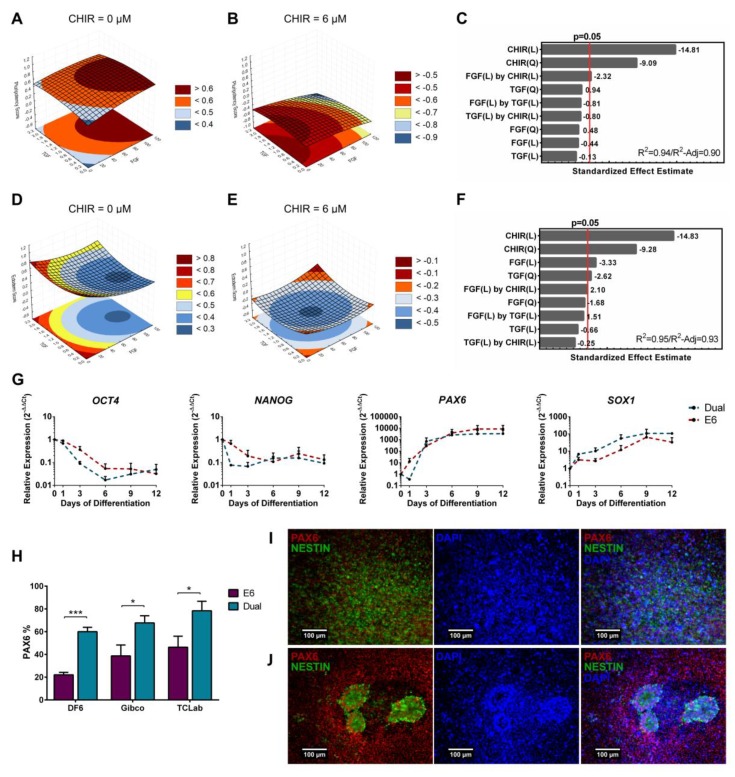
Quadratic models for the pluripotency and ectoderm scores highlighted a dominant negative contribution of Wnt signaling. (**A**,**B**) Representative curves of TGFβ and FGF2 contributions to pluripotency model with CHIR set at zero (**A**) and at 6 µM (**B**). Without CHIR, FGF2 high concentrations resulted in higher scores in the model, while with CHIR set at 6 µM, both TGFβ and FGF2 presence decreases pluripotency score. (**C**) CHIR linear and quadratic terms are the ones that contributed the most to the model, decreasing pluripotency scores. A statistically significant negative synergy can be seen between CHIR and FGF2. Model showed a good fit with a R^2^ of 0.94 and a R^2^-Adjusted of 0.90. (**D**,**E**) Representative curves of TGFβ and FGF2 contributions to ectoderm model with CHIR set to zero (**D**) and to 6 µM (**E**). Without CHIR, the model output higher ectoderm scores, concordant with the significant negative effect. (**F**) Besides CHIR linear and quadratic negative effects, FGF2 linear, TGFβ quadratic and an interaction between CHIR and FGF2 contributed significantly to the model. FGF2 positively contributed when conjugated with CHIR, while negatively contributed for ectoderm score without CHIR. TGFβ negative quadratic term contribution can be clearly observed when FGF2 is zero (**D**) with higher ectoderm scores at full or no activation. Model showed a good fit to the data set with a R^2^ of 0.95 and a R^2^-Adjusted of 0.93. (**G**) Real-time PCR comparison of E6 and dual SMAD inhibition neuroectoderm differentiation revealed a delayed expression decrease of the pluripotency markers *OCT4* and *NANOG* for the differentiation without factors (E6). Error bars, SEM, *n* = 3. (**H**) PAX6 positive cells quantification showed that dual SMAD differentiation originated 20% to 40% more PAX6^+^ cells for the three cell lines tested, when compared with E6 differentiation at day 12. Error bars, SEM, *n* = 7 DF6 and Gibco, and *n* = 5 TCLab. * *p*-value < 0.05, *** *p*-value < 0.001 (two-sided *t*-test). (**I**,**J**) At day 12, dual SMAD (**I**) differentiation did not result in neural rosette formation while cells differentiated using only E6 (**J**) consistently organized in neural rosettes throughout the culture. Scale bar: 100 µm. See Figures S2 and S3, and Tables S2 and S3 for pluripotency and ectoderm full model information.

For visualizing the true impact of Wnt signaling in each score and discern if any synergies between signaling pathways were present, full quadratic models were fitted to the data, including quadratic, linear and two-way interactions. Contribution of each factor was considered statistically significant for *p*-values < 0.05. As expected, CHIR supplementation contributed negatively for pluripotency scores (Figure 4A–C). Additionally, another significant contribution highlighted by the model could be observed, with synergy of FGF2 and CHIR contributing to lower pluripotency scores. Similar negative effects of CHIR supplementation were observed for ectoderm scores (Figure 4D–F). Furthermore, the FGF2 linear term and the TGFβ quadratic term also contributed negatively to ectoderm scores. This result is in line with FGF2 showing to repress *PAX6* [16] and TGF inhibition facilitating neuroectoderm differentiation [55]. Contrarily, a synergy of FGF2 and CHIR contributed to higher ectoderm scores, which is coherent with reports showing that this synergy can lead to ectodermal neural crest and placode lineages [30].

To further explore the differences between ectoderm induction and pluripotency maintenance highlighted in our models, hiPSC were differentiated using a combination of dual SMAD inhibitors [55], ensuring inhibition of BMP and TGFβ autocrine stimulation, and compared with cells differentiated in E6 medium only, therefore allowing hiPSCs to follow their inner circuitry and autocrine path [36]. Cells with no inhibitors (E6) showed similar profiles compared with neural differentiation induced with inhibitors (Dual), although showing a slight delay in the decrease of pluripotent markers *OCT4* and *NANOG*, and in the increase of *SOX1* (Figure 4G). This was reflected in PAX6^+^ cells originated at day 12 for all three cell lines tested, with dual SMAD inhibition resulting in a 20% to 40% increase in neural progenitors (Figure 4H,I). Nevertheless, cell differentiated in E6 medium only originated significant amounts of PAX6^+^ cells as well (Figure 4H). In fact, multiple neural rosettes were observed at day 12 for all the three hiPSC cell lines differentiated in E6 (Figure 4J), suggesting that this condition can allow a high degree of neural progenitor organization and commitment (Figure 4I) [36]. These results show the natural tendency for hiPSCs to converge to ectoderm if not actively stimulated [36,62], and are in line with the signaling inputs contributing significantly for both models.

### 3.4. Full Quadratic Models for the Mesendoderm and Mesoderm Lineage Scores

**Figure 5:**
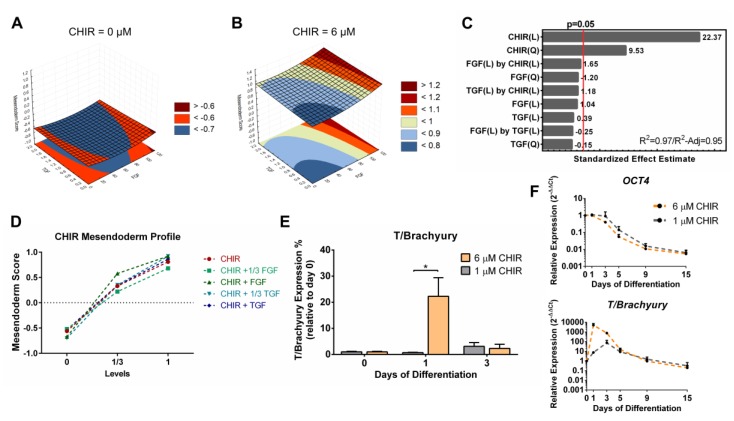
Quadratic model for the mesendoderm scores highlighted a strong positive contribution of Wnt signaling. (**A**,**B**) Representative curves of TGFβ and FGF2 contributions to the mesendoderm model with CHIR set to zero (**A**) and to 6 µM (**B**). (**C**) CHIR linear and quadratic terms of the model significantly contributed to the mesendoderm model. The model showed a very good fit to the data set, with a R^2^ of 0.97 and a R^2^-Adjusted of 0.95. (**D**) Mesendoderm score profile of CHIR supplemented conditions shows an increase with CHIR concentration. (**E**) Flow cytometry of T/Brachyury showed that expression is significantly higher when 6 µM of CHIR is used compared to a lower activation level (1 µM). Error bars, SEM, *n* = 3. * *p*-value < 0.05 (two-sided *t*-test). (**F**) Real-time PCR comparison of *OCT4* and *T*/*Brachyury* for cardiac differentiation using 6 µM or 1 µM of CHIR showed a similar decreasing profile of OCT4 gene expression, while 6 µM of CHIR contributed to a significantly higher gene expression of T/Brachyury at day 1. Error bars, SEM, *n* = 3. See Figure S4 and Table S4 for mesendoderm full model information.

For the mesendoderm score model (Figure 5A–C), an inverse contribution from CHIR linear and quadratic terms was observed when compared with the pluripotency and ectoderm models, concordant with the results showed by the hierarchical clustering and PCAs. CHIR terms were the only components of the model that were statistically significant (Figure 5C). Input of 1/3 (2 µM) of CHIR registered a steep increase in mesendoderm scores with the full input doubling the score (Figure 5D). To further understand and validate the mesendoderm model, cardiac differentiation was performed using the full level of CHIR (6 µM) and compared with a low level of Wnt Activation (1 µM), since hiPSCs died in the differentiation conditions without CHIR. Only 6 µM of CHIR contributed significantly for the expression of the mesendoderm transcription factor T/Brachyury with a peak at day 1 of both protein (Figure 5E) and mRNA (Figure 5F), despite both conditions contributing similarly to a decrease in *OCT4* (Figure 5F). The observation that 1 µM of CHIR is insufficient for the entrance into mesendoderm is in full concordance with the model obtained, which predicts negative mesendoderm scores for that level of Wnt activation with or without the activation of TGF and FGF signaling (Figure S4).

**Figure 6:**
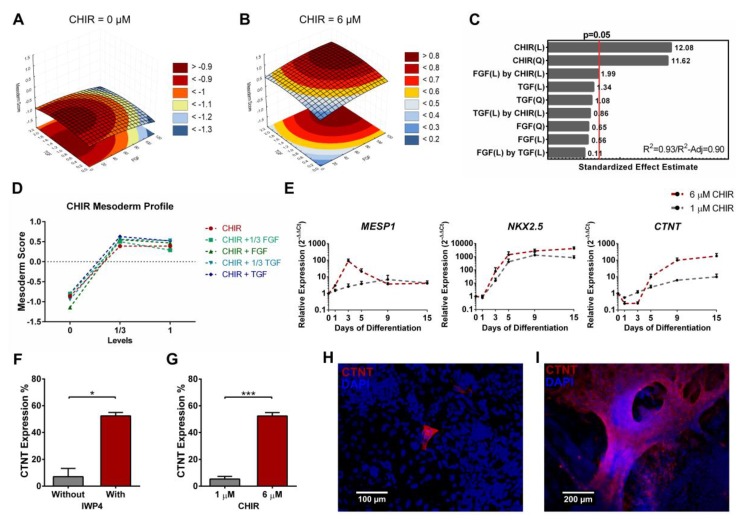
Quadratic model for the mesoderm scores highlighted the contribution of Wnt signaling with higher scores for intermediate CHIR concentrations. (**A**,**B**) Representative curves of TGFβ and FGF2 contributions to the mesoderm model with CHIR set at zero (**A**) and at 6 µM (**B**). (**C**) CHIR quadratic and linear terms significantly contributed to the mesoderm model. A synergy of CHIR with FGF can also be observed. Model showed a good fit to the data set with a R^2^ of 0.93 and a R^2^-Adjusted of 0.90. (**D**) CHIR mesoderm profile shows an increase in mesoderm score at 1/3 activation, while higher concentrations maintain or slightly decrease mesoderm scores. (**E**) Real-time PCR comparison of cardiac differentiation using 6 µM or 1 µM of CHIR registered a higher gene expression of *MESP1*, with a peak at day 3, *NKX2.5* and *CTNT* when 6 µM is used. Error bars, SEM, *n* = 3. (**F**) Flow cytometry of cardiac differentiation with or without IWP4 showed that inhibiting Wnt signaling at day 3 is essential to efficiently obtain CTNT positive cells. Error bars, SEM, *n* = 3. * *p*-value < 0.05 (two-sided *t*-test). (**G**) Flow cytometry comparing Wnt signaling low or high activation levels showed that initial low activation originated few CTNT positive cells compared to 6 µM of CHIR. Error bars, SEM, *n* = 3. *** *p*-value < 0.001 (two-sided *t*-test). (**H**,**I**) Consistent with flow cytometry, immunostaining showed that 1 µM of CHIR (H, scale bar 100 µm) originated few cardiomyocytes while 6 µM originated cardiomyocytes throughout the culture (I, scale bar 200 µm). See Figure S5 and Table S5 for mesoderm full model information.

Similarly, CHIR linear and quadratic terms also contributed significantly for the mesoderm score model (Figure 6A–C). In addition, FGF and CHIR linear terms present a positive synergy in the cutoff of statistical significance for the model (*p* = 0.06), which can be observed in Figure 6B. This synergy is in agreement with reports showing that dual activation of FGF and Wnt promotes hiPSC differentiation into mesenchymal stem cells [63] and the generation of neuromesodermal progenitors [64,65]. FGF signaling also had a positive contribution for endoderm scores, with the FGF linear term and both CHIR quadratic and linear terms having a significant positive impact in the scores (Figures S6 and S7). This is in line with reports showing that both factors are essential to efficient definitive endoderm differentiation [31], and with FGF signaling pathway playing an important role in further differentiating the definitive endoderm, particularly into liver, lung and pancreatic lineages [18,59,66].

Contrarily to the mesendoderm model, stimulation with 2 µM of CHIR gave rise to the higher mesoderm scores, while the full input of 6 µM disclosed a tendency to stagnate or even decrease such scores (Figure 6D). For the generation of cardiac mesoderm, stimulation with 6 µM of CHIR resulted in increased *MESP1* expression, with a peak at day 3. Furthermore, *NKX2.5* expression was not significantly affected by the level of Wnt signaling stimulation, but *CTNT* expression was one order of magnitude higher at 6 µM when compared with 1 µM of CHIR (Figure 6E). Concomitant with our model prediction, later inhibition of Wnt signaling, using IWP4 [27], was fundamental to obtain hiPSC-derived cardiomyocytes (Figure 6F), an observation that can be predicted in our model by the maintenance or even decrease in mesoderm scores when a full level input of CHIR was used (Figure 6D and Figure S5). Further aligned with the model predictions, low activation of Wnt signaling originated a low number of cardiomyocytes (Figure 6G), particularly sparse and rarely observable (Figure 6H) when compared to the full level of input (Figure 6I).

## 4. Discussion

**Figure 7:**
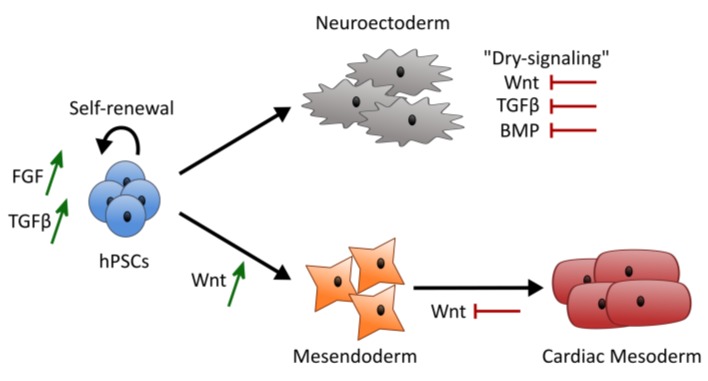
Model summarizing the overall results obtained using a dry-signaling multiparameter approach. Wnt signaling activation showed to be dominant over FGF and TGFβ signaling driving hPSCs towards mesendoderm lineages. A synergy of FGF and CHIR was observed providing higher ectoderm scores or higher mesoderm scores and contributing to lower pluripotency scores. Contribution of FGF and TGFβ signaling to maintain pluripotency scores seems to be connected with the negative contribution of both FGF and TGFβ signaling to ectoderm scores, with absence of inputs inducing cells to follow their inner circuitry towards neuroectoderm.

There is a multitude of signaling pathways and interactions that govern pluripotency maintenance and lineage specification. Uncontrolled and poorly defined systems with increased noise to signal ratio hinder the ability to fully understand the independent and synergistic role of each factor in hPSC fate. The focus of our study was to develop a multiparameter approach to study the individual and synergistic effect of Wnt, FGF and TGFβ signaling pathways using a dry-signaling culture system to avoid major unspecific signaling contributions. Our results showed that Wnt signaling had a dominant effect over FGF and TGFβ inputs, pulling hiPSCs away from pluripotency and ectoderm, towards mesendoderm lineages. In addition, a synergy between FGF and Wnt signaling was observed, with a negative contribution to pluripotency scores and a positive contribution to ectoderm and mesoderm scores. FGF and TGFβ signaling negatively contributed to ectoderm scores, which is connected with the well-known role of these signaling pathways in maintaining hiPSCs epiblast-like pluripotent state [4,15], and preventing cells to follow their inner circuitry towards neuroectoderm [36,62] (Figure 7).

In our model, the linear contribution of FGF and the quadratic term of TGFβ show a negative correlation to the ectoderm score, with the lowest result obtained for levels of 100 ng/mL of FGF (full input) and 0.7 ng/mL of TGFβ (1/3 of input). These values are in concordance with the level of input provided by both commercial media E8 and TeSR used to maintain hiPSCs pluripotency [15,42]. In addition, these levels of input prevent hiPSCs to naturally exit pluripotency towards neuroectoderm as observed by us and previously reported by Lippmann and coworkers [36], and are in concordance with reports showing that both factors inhibit neuroectoderm differentiation [16,55]. As suggested from our results, the role of FGF and TGFβ in pluripotency maintenance seems to derive from a thin balance that prevents exit towards differentiation, instead of actively promoting and stimulating pluripotency (Figure 4). This seems to be in line with the weak stability of the pluripotent epiblast-like state [7,8], and the bias of hPSCs towards neuroectoderm when only both factors are present to maintain pluripotency [36,62].

In the presence of Wnt signaling stimulation, TGF and FGF signaling effects were secondary, with all models showing CHIR terms as the most significant contributors. This dominance contributed negatively for pluripotency and ectoderm scores (Figure 4), and positively for mesendoderm, mesoderm and endoderm scores (Figure 5 and Figure 6, Figures S5 and S6). These results are coherent with the literature and the well-known importance of Wnt signaling in the commitment of hPSCs towards mesendoderm and lineage specifications that rely on Wnt activation, such as cardiac mesoderm [27], pancreatic β-cells [67], mesenchymal stem cells [63], or epicardial lineage cells [29]. Besides contributing to commitment, Wnt signaling is described to have a role in promoting self-renewal and the naïve state of pluripotency [9,10,11,34]. Our pluripotency model predicted higher pluripotency scores for input levels lower than 2 μM, but was unable to fully register a positive contribution of Wnt signaling to pluripotency. This might be explained by the epiblast-like state of the hiPSCs used in our study, and the inability of Wnt activation to reprogram cells to the naïve state by itself [10,11,49]. Once in a primed state, Wnt signaling role seems to transition from self-renewal to promoting further commitment to mesendoderm [23].

Synergies observed between Wnt signaling and FGF signaling in our models are also coherent with previously reported data. The positive contribution for the ectoderm model is in line with the role of FGF in repressing *PAX6* [16] and, together with Wnt, synergistically promoting the specification towards ectodermal neural crest and placode lineages [26,30]. The proximity between the pluripotent state with neuroectoderm specification can explain the negative synergistic impact of both pathways in the pluripotency model. In addition, contribution to lower pluripotency scores is also in line with the impact of Wnt in promoting mesendoderm lineages, and FGF being important to specify mesendoderm towards endoderm lineages, which is also coherent with the predictions of our endoderm model [18,31,59,66,68]. Lastly, the synergistic effect of both pathways in the mesoderm model is in line with the paraxial specification and direct differentiation of hiPSCs towards mesenchymal and neuromesodermal progenitors [32,63,64,65].

In conclusion, using a multifactorial, multiparameter modeling approach we predicted a dominant role of Wnt signaling over FGF and TGF signaling in our dry-signaling culture system. This modeling methodology also allowed the construction of models providing a rational understanding of hiPSCs pluripotency and commitment, allowing to discriminate the different synergies between FGF and Wnt signaling, in agreement with previously reported studies. Following this proposed framework, carefully designed 5-level fractional factorial designs coupled with multiple signaling activation dynamics should contribute to the construction of models with increased sensitivity and reduced variance and, consequently, providing an extra degree of comprehension of the complex regulatory system of human pluripotency and commitment.

## Figures and Tables

**Table 1:** Full factorial design conditions. FGF2 concentration levels ranged between 0, 35, and 100 ng/mL; TGFβ concentration levels ranged between 0, 0.85, and 2 ng/mL; and CHIR concentration levels ranged between 0, 2, and 6 µM.

Samples	FGF2 (ng/mL)	TGFβ (ng/mL)	CHIR (µM)
Sample 1/E6	0	0	0
Sample 2	0	0	2
Sample 3	0	0	6
Sample 4	0	0.7	0
Sample 5	0	0.7	2
Sample 6	0	0.7	6
Sample 7	0	2	0
Sample 8	0	2	2
Sample 9	0	2	6
Sample 10	35	0	0
Sample 11	35	0	2
Sample 12	35	0	6
Sample 13	35	0.7	0
Sample 14	35	0.7	2
Sample 15	35	0.7	6
Sample 16	35	2	0
Sample 17	35	2	2
Sample 18	35	2	6
Sample 19	100	0	0
Sample 20	100	0	2
Sample 21	100	0	6
Sample 22	100	0.7	0
Sample 23	100	0.7	2
Sample 24	100	0.7	6
Sample 25	100	2	0
Sample 26	100	2	2
Sample 27	100	2	6

## Supplementary Materials

The following are available online at https://www.mdpi.com/2306-5354/6/3/71/s1, Table S1: Primer pairs used for real-time PCR, Table S2: ANOVA Pluripotency score model, Table S3: ANOVA Ectoderm score model, Table S4: ANOVA Mesendoderm score model, Table S5: ANOVA Mesoderm score model, Table S6: ANOVA Endoderm score model, Figure S1: Cell morphology changes during cocktail exposure, Figure S2: Full panel of the quadratic model for the pluripotency scores highlighting a dominant negative contribution of Wnt signaling, Figure S3: Full panel of the quadratic model for the ectoderm scores highlighting a dominant negative contribution of Wnt signaling with FGF signaling also contributing to lower ectoderm scores, Figure S4: Full panel of the quadratic model for the mesendoderm scores highlighting a strong and dominant contribution of Wnt signaling, Figure S5: Full panel of the quadratic model for the mesoderm scores highlighting the contribution of Wnt signaling with higher scores for intermediate CHIR concentrations, Figure S6: Full panel of the quadratic model for the endoderm scores highlighted the contribution of Wnt signaling with FGF signaling also positively contributing to higher endoderm scores, Figure S7: Endoderm Model Score profiles and Standardized Effect Estimate.
