# Supplementary material for: Multifactorial Modeling Reveals a Dominant Role of Wnt Signaling in Lineage Commitment of Human Pluripotent Stem Cells
Source: Bioengineering (Basel). 2019 Aug 15;6(3):71. doi: 10.3390/bioengineering6030071 (PMC6783940; doi:10.3390/bioengineering6030071)
Supplement: Supplementary file 1 [file bioengineering-06-00071-s001.pdf]

Supplementary Information

Dias *et al.* 2019

Table S1. Primer pairs used for real-time PCR.

Gene	Primers (5′ > 3′)	bp	Tm (ºC)	Amplicon Size
OCT4	Fwd: GAGAACCGAGTGAGAGGCAACC	22	62.9	166
	Rev: CATAGTCGCTGCTTGATCGCTTG	23	62.4	
NANOG	Fwd: AATACCTCAGCCTCCAGCAGATG	23	62.1	149
	Rev: TGCGTCACACCATTGCTATTCTTC	24	61.9	
FGF5	Fwd: CACTGATAGGAACCCTAGAGGC	22	61.0	196
	Rev: CAGATGGAAACCGATGCCC	19	60.5	
PAX6	Fwd: AGTGCCCGTCCATCTTTGC	19	62.6	81
	Rev: CGCTTGGTATGTTATCGTTGGT	22	60.7	
P75	Fwd: ATCCTGGCTGCTGTGGTTGT	20	62.1	158
	Rev: TCCACGGAGATGCCACTGTC	20	61.9	
T	Fwd: CTATTCTGACAACTCACCTGCAT	23	60.0	146
	Rev: ACAGGCTGGGGTACTGACT	19	61.9	
MIXL1	Fwd: TACCCCGACATCCACTTGCG	20	62.2	110
	Rev: CCACTCTGACGCCGAGACTT	20	61.9	
MESP1	Fwd: CTGAAGGGCAGGCGATGGA	19	62.0	83
	Rev: GGGCATCCAGGTCTCCAACA	20	61.9	
NKX2.5	Fwd: CCAAGGACCCTAGAGCCGAA	20	61.0	77
	Rev: GTCCGCCTCTGTCTTCTCCA	20	61.3	
PDX1	Fwd: CACCTTCACCACCACCTCCC	20	62.4	207
	Rev: CGTCCGCTTGTTCTCCTCCG	20	63.1	
SOX17	Fwd: CTCCGGTGTGAATCTCCCC	19	59.5	94
	Rev: CACGTCAGGATAGTTGCAGTAAT	23	58.6	
GAPDH	Fwd: ACAACTTTGGTATCGTGGAAGG	22	60.2	101
	Rev: GCCATCACGCCACAGTTTC	19	61.7	

Melting temperatures (Tm), amplicon sizes and primer specificity were estimated using Primer Blast.

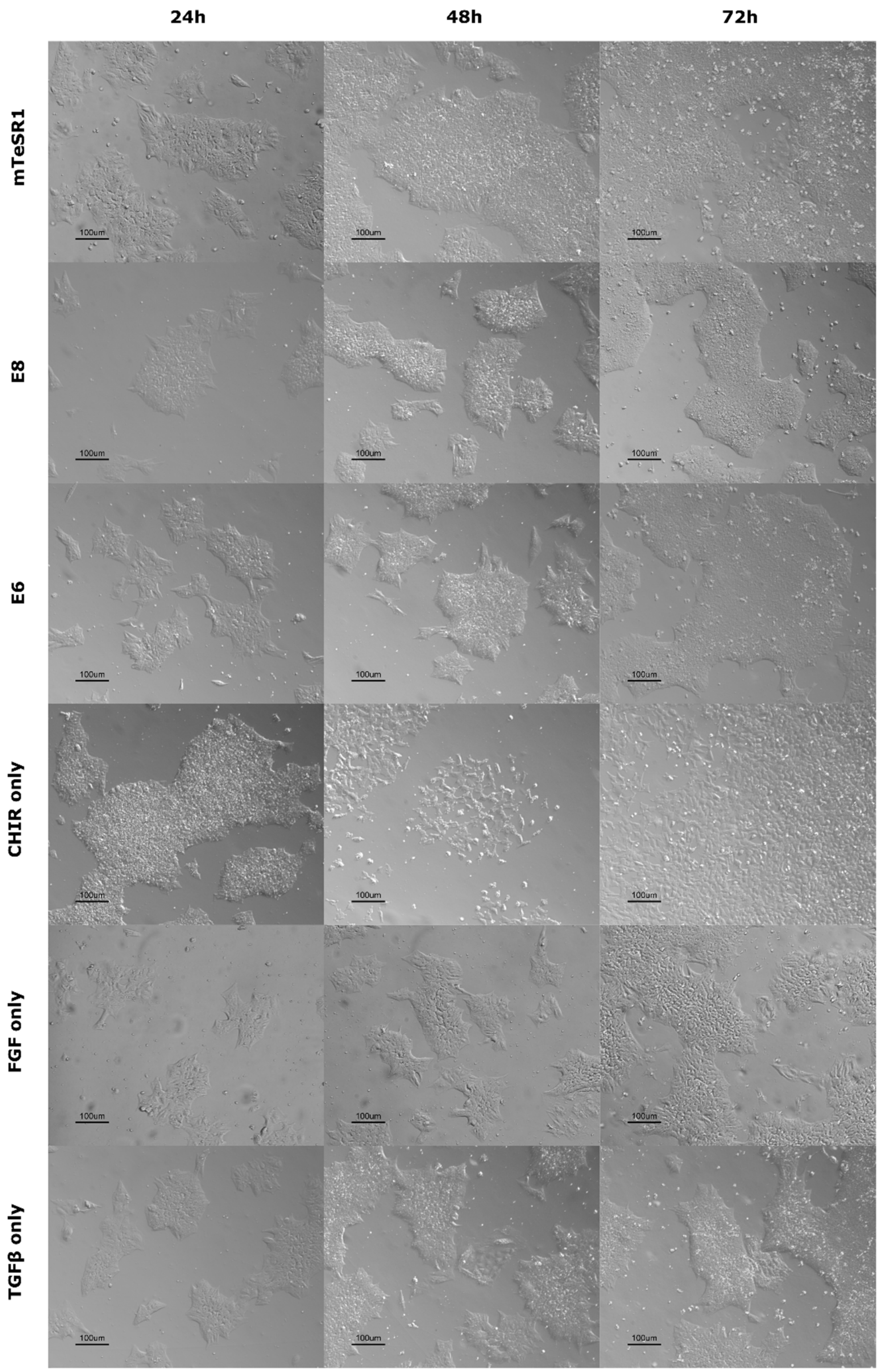

Figure S1. Cell morphology changes during cocktail exposure.

Condition with CHIR only shows a gradual change in cell morphology over exposure time, typically associated with a more differentiated-like phenotype, different of the morphology observed for controls and cocktails with TGF and/or FGF without CHIR, with more well-defined compact colonies, typically associated with the pluripotent state. Cell death was not increased when compared to controls, FGF only and TGFβ only at any time point.

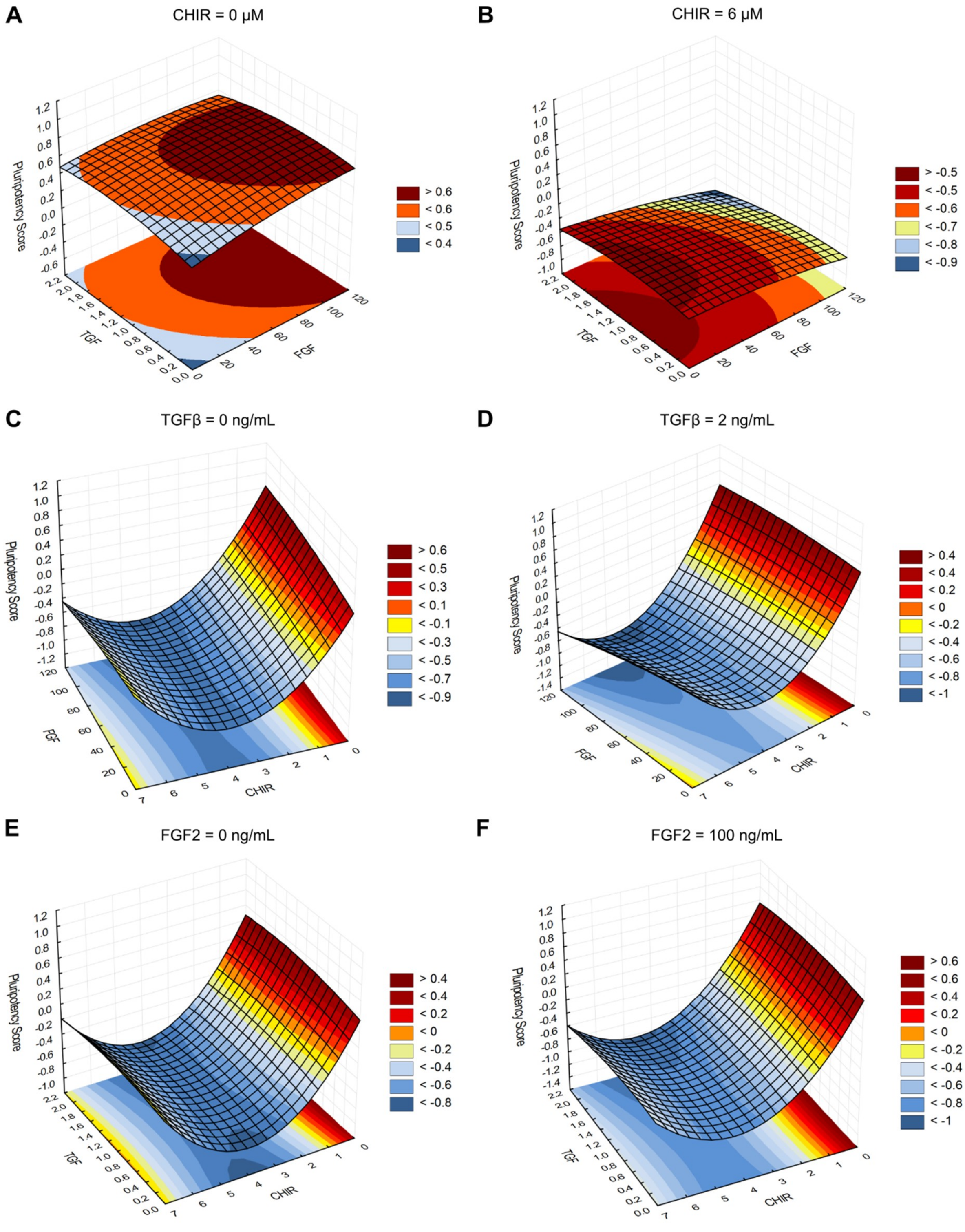

Figure S2. Full panel of the quadratic model for the pluripotency scores highlighting a dominant negative contribution of Wnt signaling.

Top, TGFβ and FGF2 contributions to the pluripotency model with CHIR set to zero (A) and to 6 μM (B). Middle, FGF2 and CHIR contributions to the pluripotency model with TGFβ set at zero (C) and at 2 ng/mL (D). Bottom, TGFβ and CHIR contributions to the pluripotency model with FGF2 set at zero (E) and at 100 ng/mL (F). Overall, model shows a negative contribution of Wnt activation to pluripotency score. An FGF and CHIR synergy is also contributing to lower pluripotency scores.

Table S2. ANOVA Pluripotency score model.

	SS	df	MS	F	p-value
(1) FGF (L)	0.005	1	0.005	0.194	0.664
FGF (Q)	0.006	1	0.006	0.228	0.639
(2) TGF (L)	0.000	1	0.000	0.017	0.897
TGF (Q)	0.024	1	0.024	0.890	0.357
**(3) CHIR (L)**	**6.010**	**1**	**6.010**	**219.264**	**6.9 x 10^{-12}**
**CHIR (Q)**	**2.262**	**1**	**2.262**	**82.545**	**2.4 x 10^{-08}**
1L by 2L	0.018	1	0.018	0.656	0.428
**1L by 3L**	**0.148**	**1**	**0.148**	**5.402**	**0.031**
2L by 3L	0.017	1	0.017	0.632	0.436
Error	0.521	19	0.027		
Total SS	8.013	28			

SS (Sum of Squares); df (Degree of Freedom); MS (Mean of Squares); F (Fisher's Statistical test). In bold, statistically significant factors with p-values lower than 0.05.

Table S3. ANOVA Ectoderm score model.

	SS	df	MS	F	p-value
**(1) FGF (L)**	**0.174**	**1**	**0.174**	**11.066**	**0.004**
FGF (Q)	0.044	1	0.044	2.823	0.109
(2) TGF (L)	0.007	1	0.007	0.433	0.518
**TGF (Q)**	**0.108**	**1**	**0.108**	**6.880**	**0.017**
**(3) CHIR (L)**	**3.466**	**1**	**3.466**	**219.985**	**6.7 x 10^{-12}**
**CHIR (Q)**	**1.355**	**1**	**1.355**	**86.031**	**1.7 x 10^{-08}**
1L by 2L	0.036	1	0.036	2.291	0.147
**1L by 3L**	**0.070**	**1**	**0.070**	**4.425**	**0.049**
2L by 3L	0.001	1	0.001	0.061	0.808
Error	0.299	19	0.016		
Total SS	6.242	28			

SS (Sum of Squares); df (Degree of Freedom); MS (Mean of Squares); F (Fisher's Statistical test). In bold, statistically significant factors with p-values lower than 0.05.

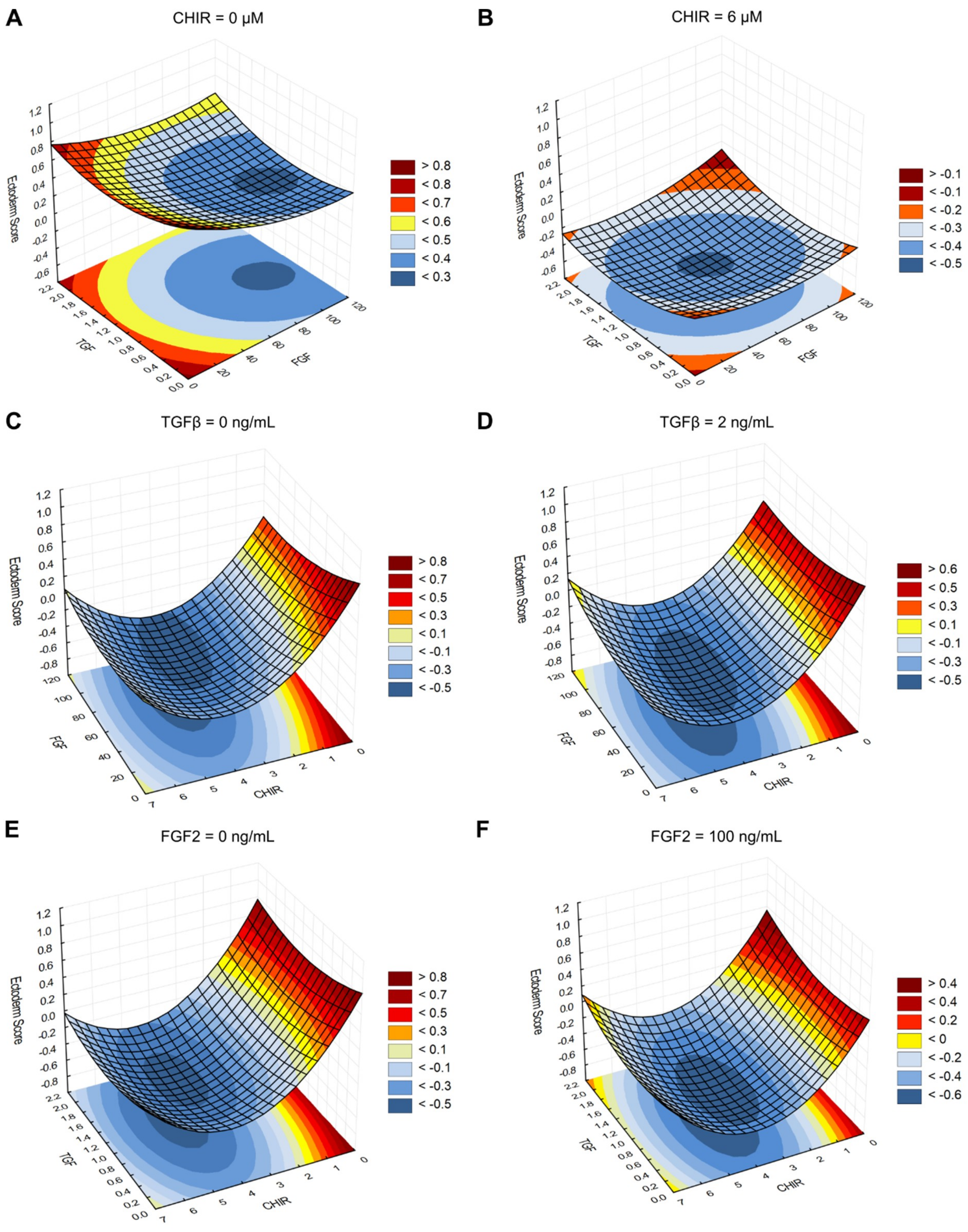

Figure S3. Full panel of the quadratic model for the ectoderm scores highlighting a dominant negative contribution of Wnt signaling with FGF signaling also contributing to lower ectoderm scores.

Top, TGFβ and FGF2 contributions to the ectoderm model with CHIR set to zero (A) and to 6 µM (B). Middle, FGF2 and CHIR contributions to the ectoderm model with TGFβ set at zero (C) and at 2 ng/mL (D). Bottom, TGFβ and CHIR contributions to the ectoderm model with FGF2 set at zero (E) and at 100 ng/mL (F). Overall, model shows a negative contribution of Wnt activation to ectoderm score. TGF and FGF signaling contributed negatively, while a synergy of CHIR and FGF contributed positively for ectoderm scores.

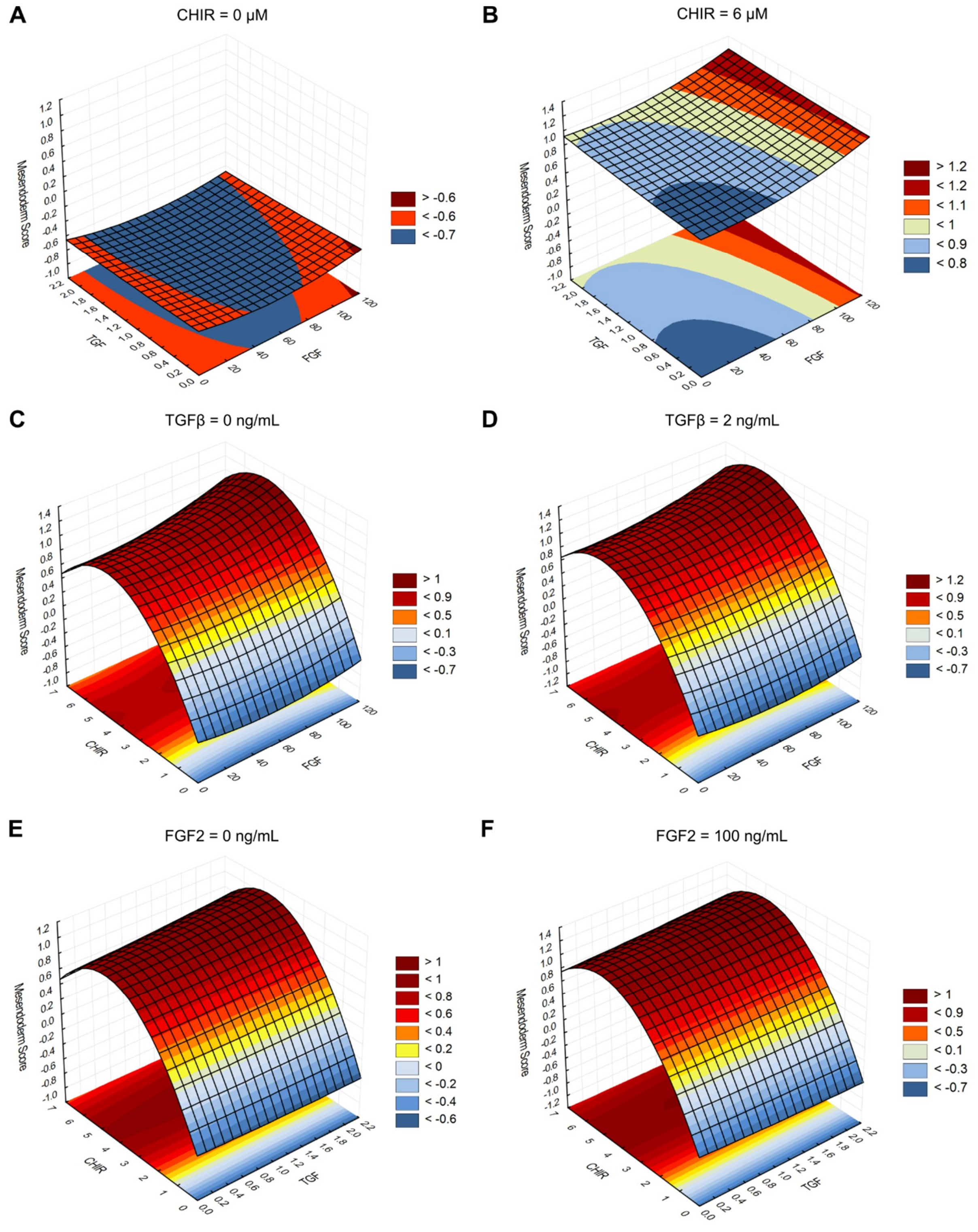

Figure S4. Full panel of the quadratic model for the mesendoderm scores highlighting a strong and dominant contribution of Wnt signaling.

Top, TGFβ and FGF2 contributions to the mesendoderm model with CHIR set to zero (A) and to 6 µM (B). Middle, FGF2 and CHIR contributions to the mesendoderm model with TGFβ set at zero (C) and at 2 ng/mL (D). Bottom, TGFβ and CHIR contributions to the mesendoderm model with FGF2 set at zero (E) and at 100 ng/mL (F). Overall, model shows a positive contribution of Wnt activation to mesendoderm score. Model predicts that CHIR 1 µM contributes for negative mesendoderm scores.

Table S4. ANOVA Mesendoderm score model.

	SS	df	MS	F	p-value
(1) FGF (L)	0.025	1	0.025	1.089	0.310
FGF (Q)	0.033	1	0.033	1.445	0.244
(2) TGF (L)	0.003	1	0.003	0.150	0.703
TGF (Q)	0.001	1	0.001	0.023	0.881
**(3) CHIR (L)**	**11.565**	**1**	**11.565**	**500.279**	**4.1×10^{-15}**
**CHIR (Q)**	**2.099**	**1**	**2.099**	**90.812**	**1.1×10^{-08}**
1L by 2L	0.001	1	0.001	0.061	0.807
1L by 3L	0.063	1	0.063	2.711	0.116
2L by 3L	0.032	1	0.032	1.389	0.253
Error	0.439	19	0.023		
Total SS	13.627	28			

SS (Sum of Squares); df (Degree of Freedom); MS (Mean of Squares); F (Fisher's Statistical test). In bold, statistically significant factors with p-values lower than 0.05

Table S5. ANOVA Mesoderm score model.

	SS	df	MS	F	p-value
(1) FGF (L)	0.025	1	0.025	0.311	0.584
FGF (Q)	0.034	1	0.034	0.418	0.526
(2) TGF (L)	0.144	1	0.144	1.787	0.197
TGF (Q)	0.094	1	0.094	1.166	0.294
**(3) CHIR (L)**	**11.764**	**1**	**11.764**	**145.963**	**2.3×10^{-10}**
**CHIR (Q)**	**10.879**	**1**	**10.879**	**134.987**	**4.5×10^{-10}**
1L by 2L	0.001	1	0.001	0.013	0.911
**1L by 3L**	**0.320**	**1**	**0.320**	**3.971**	**0.061**
2L by 3L	0.060	1	0.060	0.744	0.399
Error	1.531	19	0.081		
Total SS	22.548	28			

SS (Sum of Squares); df (Degree of Freedom); MS (Mean of Squares); F (Fisher's Statistical test). In bold, statistically significant factors with p-values lower than 0.05.

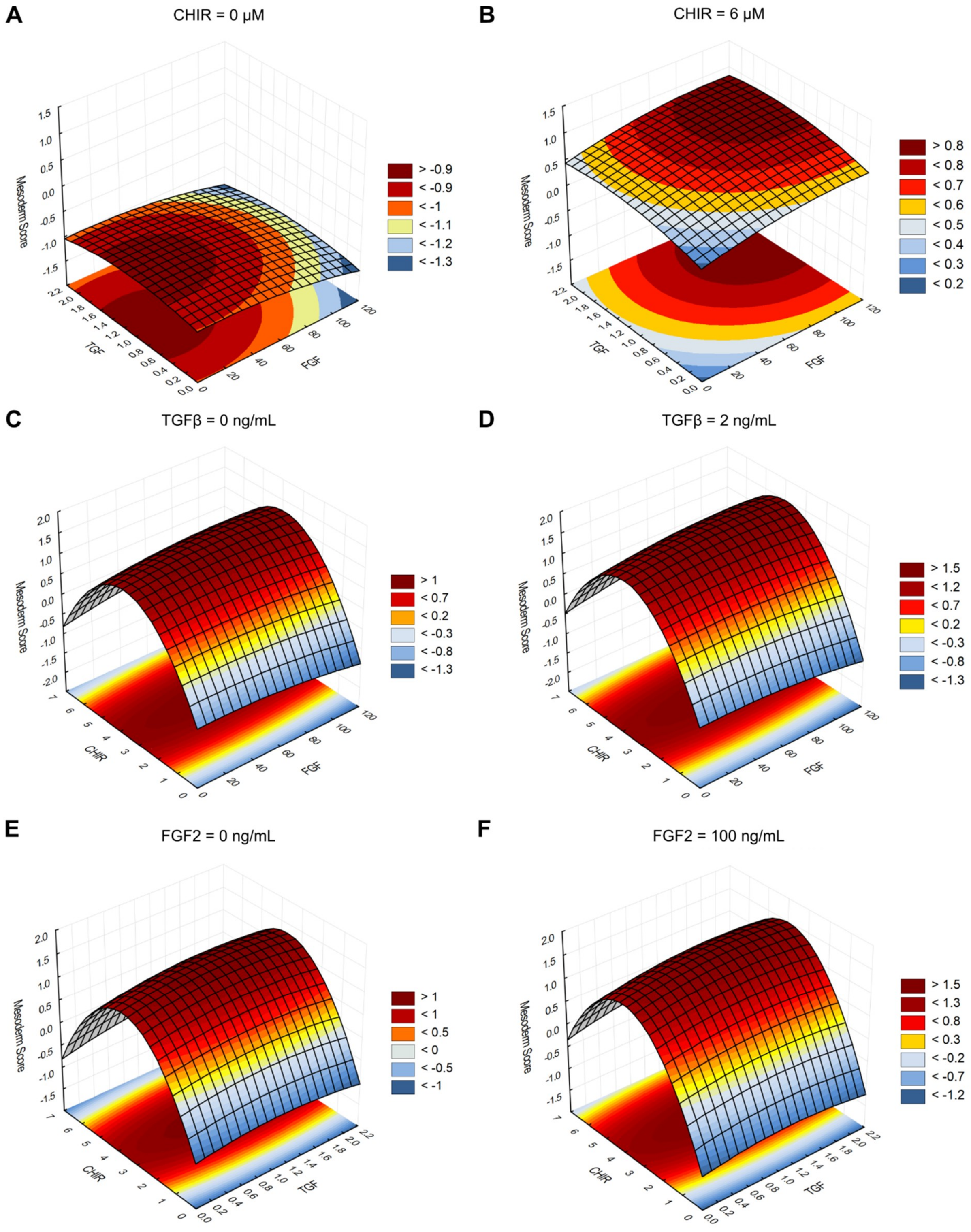

Figure S5. Full panel of the quadratic model for the mesoderm scores highlighting the contribution of Wnt signaling with higher scores for intermediate CHIR concentrations.

Top, TGFβ and FGF2 contributions to the Mesoderm model with CHIR set to zero (A) and to 6 µM (B). Middle, FGF2 and CHIR contributions to the Mesoderm model with TGFβ set at zero (C) and at 2 ng/mL (D). Bottom, TGFβ and CHIR contributions to the endoderm model with FGF2 set at zero (E) and at 100 ng/mL (F). Overall, model shows a positive contribution of Wnt activation to mesoderm scores, with scores reaching their highest value at intermediate levels of activation. When FGF2 and

TGFβ are full activated, higher concentrations of CHIR were needed to achieve the higher mesoderm score values (B).

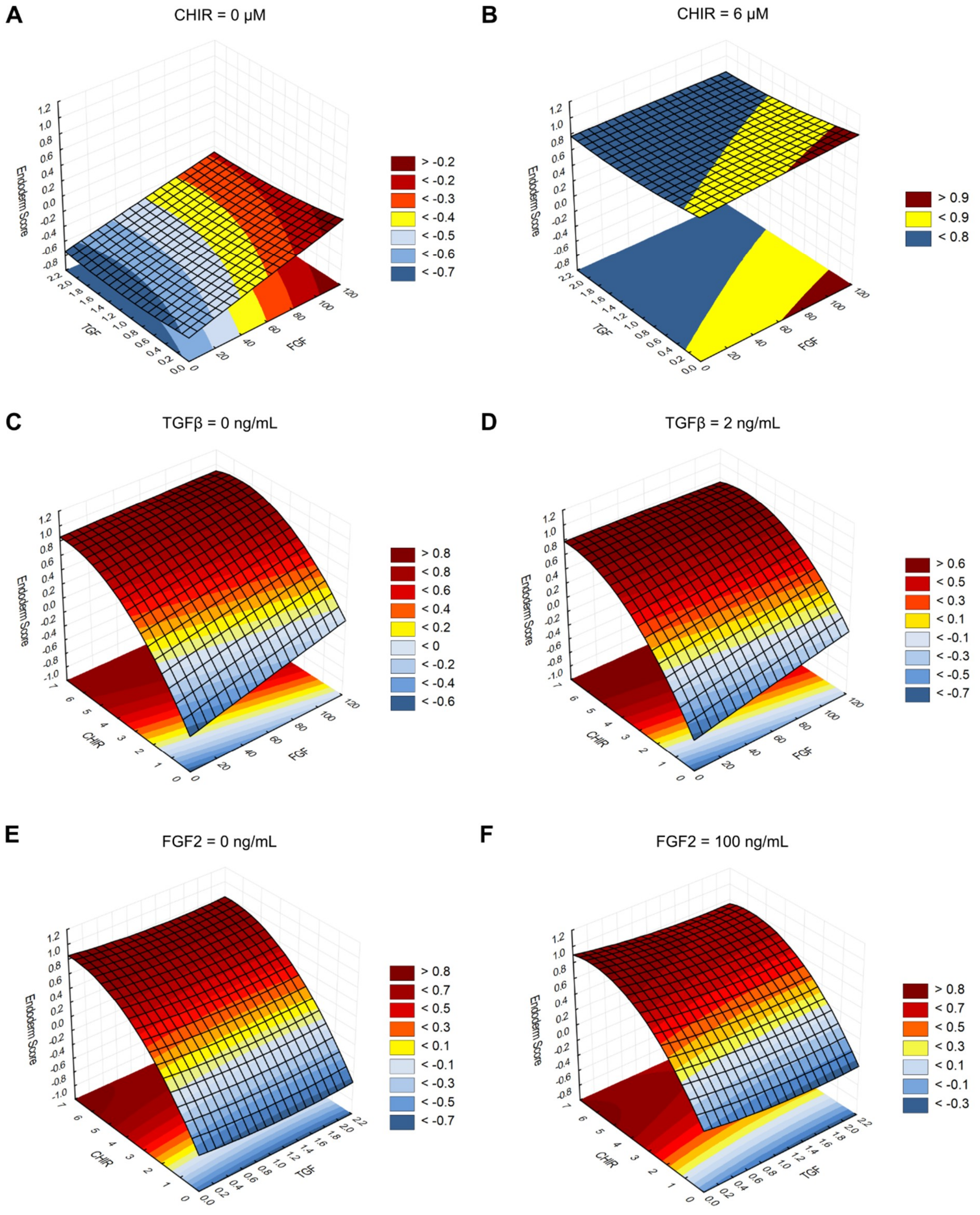

Figure S6. Full panel of the quadratic model for the endoderm scores highlighted the contribution of Wnt signaling with FGF signaling also positively contributing to higher endoderm scores.

Top, TGFβ and FGF2 contributions to the endoderm model with CHIR set to zero (A) and to 6 μM (B). Middle, FGF2 and CHIR contributions to the endoderm model with TGFβ set at zero (C) and

at 2 ng/mL (D). Bottom, TGFβ and CHIR contributions to the endoderm model with FGF2 set at zero (E) and at 100 ng/mL (F). Overall, model shows a positive contribution of Wnt activation to endoderm score, with scores continuously increasing with CHIR concentration. FGF also provided a major contribution to higher endoderm scores.

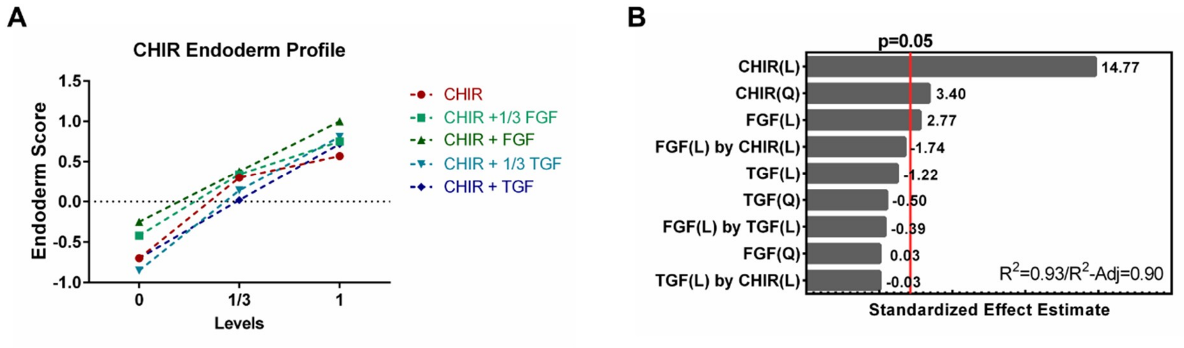

Figure S7. Endoderm Model Score profiles and Standardized Effect Estimate. (A) CHIR endoderm profile shows an increase for all CHIR supplemented conditions in endoderm scores with CHIR concentration. The higher level of CHIR input alone contributed less to the endoderm score when compared to CHIR in combination with FGF or TGF. (B) CHIR quadratic and linear terms positively contributed to the endoderm score, with FGF2 linear term also contributing to higher endoderm scores. Model showed a good fit with a R^2 of 0.93 and a R^2-Adjusted of 0.90.

Table S6. ANOVA Endoderm score model.

	SS	df	MS	F	p-value
**(1) FGF (L)**	**0.284**	**1**	**0.284**	**7.698**	**0.012**
FGF (Q)	0.000	1	0.000	0.001	0.977
(2) TGF (L)	0.055	1	0.055	1.487	0.238
TGF (Q)	0.009	1	0.009	0.251	0.622
**(3) CHIR (L)**	**8.044**	**1**	**8.044**	**218.185**	**7.2×10^{-12}**
**CHIR (Q)**	**0.425**	**1**	**0.425**	**11.540**	**0.003**
1L by 2L	0.006	1	0.006	0.154	0.700
1L by 3L	0.112	1	0.112	3.030	0.098
2L by 3L	0.000	1	0.000	0.001	0.977
Error	0.700	19	0.037		
Total SS	10.745	28			

SS (Sum of Squares); df (Degree of Freedom); MS (Mean of Squares); F (Fisher's Statistical test).
In bold, statistically significant factors with p-values lower than 0.05.
